# Personality and Smartphone Addiction in Romania’s Digital Age: The Mediating Role of Professional Status and the Moderating Effect of Adaptive Coping

**DOI:** 10.3390/jintelligence13070086

**Published:** 2025-07-15

**Authors:** Daniela-Elena Lițan

**Affiliations:** Psychology Department, West University of Timișoara, 300223 Timișoara, Romania; daniela.litan@e-uvt.ro

**Keywords:** personality factors, coping strategies, smartphone addiction, professional status

## Abstract

In this research, we aimed to evaluate the relationship between the main dimensions of personality (Extraversion, Maturity, Agreeableness, Conscientiousness, and Self-actualization) and mobile phone addiction, both directly and mediated by the professional context (professional status), and moderated by adaptive cognitive-emotional coping strategies. The participants, adult Romanian citizens, completed measures of personality—Big Five ABCD-M, a mobile phone addiction questionnaire, and the CERQ for adaptive coping strategies. They also responded to a question about current professional status (employed, student, etc.). Data were analyzed using Jamovi, and the findings were somewhat unexpected, though it aligned with the existing literature. Maturity emerged as a consistent inverse predictor of smartphone addiction (r = −0.45, β = −0.43, *p* < 0.001) across all three analyses. Extraversion showed an indirect effect mediated by professional status (β = −0.077, *p* < 0.05). Self-actualization was also found to predict smartphone addiction positively through full mediation by professional status (β = 0.05, *p* < 0.05). Agreeableness became a significant negative predictor (β = −0.13, *p* < 0.05) only when adaptive coping strategies were included. These findings highlight that the transition from frequent smartphone use—whether for work or personal reasons—to addiction can be subtle. This study may support both the general population in understanding smartphone use from a psycho-emotional perspective and organizations in promoting a healthy work-life balance.

## 1. Introduction

It is no longer a secret to anyone that agitation, stress, anxiety, and sometimes burnout are part of our lives on a regular basis, whether we are talking about Romania or another geographical part of the world. In Ireland, the United Kingdom, and Italy, over half of the respondents to a survey conducted in 2022 reported that they were experiencing stress. In addition, almost a fifth of the respondents in most of the countries surveyed reported that they suffered from depression. These emotional states are increasingly studied in relation to patterns of smartphone overuse, where digital engagement can amplify stress responses and emotional dysregulation (Elhai et al. 2020). The feelings of anxiety were the highest in Germany at nine percent (AXA 2022). According to another study also conducted in 2022 in Europe regarding the degree of exhaustion (burnout), we found out that Romania was in a leading position regarding this suffering, second place in Europe, with 67% of respondents declaring that they had experienced or had been close to burnout (Stada 2022). In the USA, things are not much different from Europe either. According to the American Psychiatric Association, the 2024 results of the annual mental health survey show that adults are feeling increasingly anxious. In 2024, 43% of adults said that they felt more anxious than in the previous year, compared to 37% in 2023 and 32% in 2022 (American Psychiatric Association 2024). From official statistical analyses we understand that the post-pandemic period, contrary to expectations, has maintained and even increased the level of stress and anxiety. According to the survey (Wiley 2024), starting with 2023, almost half of the North American students have sought more psychological counselling or mental health therapy, the reasons being the following: worsening symptoms/more help needed in the post-pandemic period, stress of school and work/professional life balance, anxiety, the desire to feel better, and depression. Moreover, recent empirical data suggests that the use of smartphones for work-related purposes after hours significantly contributes to work-life conflict and overall stress levels (Blake et al. 2024). Technology has significantly contributed to this whole “picture”. For example, in a previous study (APA 2023), we learn that 45% of American adults felt anxiety in 2023 due to the impact of technology on their daily lives. The 2022 survey (CivicScience 2022) of internet users in the US found a link between digital device addiction and stress levels, with over 60% of those who considered themselves addicted or somewhat addicted to digital technology reporting being very or almost very stressed. On the other hand, over 50% of the respondents who were not addicted to digital devices said that they did not experience stress. This finding is consistent with more recent empirical evidence. For example, Tufan et al. (2025) demonstrated a strong positive association between the fear of missing out (FoMO) and smartphone addiction, reinforcing the role of emotional and cognitive vulnerability in excessive smartphone use. This is further supported by recent findings showing that smartphone addiction is associated with increased negative emotions and reduced life satisfaction, with pessimistic coping styles acting as a key explanatory mechanism (Zhu et al. 2025). Also, Elhai et al. (2025) extend these findings, showing that FoMO mediates the link between pathological worry and both smartphone and social media addiction, and that FoMO is a stronger predictor of addictive severity than general worry. Given these findings, it becomes increasingly evident that FoMO can be conceptualized as a domain-specific form of anxiety, which intensifies compulsive smartphone engagement.

Other findings underline that smartphone usage itself can exacerbate emotional strain; for instance, prolonged screen time and constant connectivity have been identified as predictors of anxiety, burnout, and sleep disturbances (Devi and Singh 2023; Gonçalves and Santos 2022; Stanković et al. 2021).

The use of digital devices in relation to professional activity is no longer a choice; it is an obligation and will continue to be one. Digital transformation, or the adoption of digital technology meant to transform business processes and services from non-digital to digital (moving data to the cloud using technological devices and tools for communication and collaboration, and automating processes) is in full swing, and the COVID-19 pandemic has contributed to increasing the pace of this transformation, estimating that by 2027, global spending on digital transformation will reach 3.9 trillion US dollars (Statista 2024). This explains, at least partially, the levels of stress, anxiety, depression, and burnout described by statistics as being higher today than before 2020. Working from home, having a permanent connection to the workplace facilitated by technology (laptop, smartphone, and other types of devices) anytime and anywhere affects one’s personal life (Seibert et al. 2021), and this context often turns habit into technology addiction. Furthermore, recent empirical research supports the relevance of personality factors in this dynamic. Psychological vulnerabilities such as stress, anxiety, and burnout rarely act in isolation, and are often intensified by the pervasive role of mobile technology in everyday routines, as previously discussed. In this context, Bhayangkara et al. (2024) showed that Conscientiousness and Extraversion are significantly associated with smartphone addiction in young adults.

In the following two sections, we examine the five-factor Model of Personality in relation to smartphone technology use and its moderated mediation by professional status and adaptive coping strategies.

## 2. Five-Factor Personality and Smartphone Technology Addiction

The term personality is intended to provide a sense of continuity, stability, or consistency in what a person does, thinks, or experiences (Wrosch and Scheier 2003) and it is often conceptualized as being based on five traits (Neal and Brutzman 2023):

- Openness to Experience—a trait characterized by active search, love of new experiences (Akbari et al. 2023), creativity, and esthetic appreciation (Lyon et al. 2021);
- Conscientiousness—a trait characterized by an orderly and diligent approach to completing tasks and goals (Williams et al. 2023);
- Extraversion—a trait characterized by a tendency to be assertive and sociable (Kang 2023);
- Agreeableness—a trait characterized by compassion toward others, rather than antagonism (Skoglund et al. 2020) and politeness (Lyon et al. 2021);
- Neuroticism—a trait also known as emotional instability and characterized by stress, anxiety, impulsivity, depression, anger, and vulnerability (Bano et al. 2019).

Five-factor theory posits that these broad trait domains emerge from basic psycholexical structures and stable biological predispositions (Allport and Odbert 1936; Fiske 1949; Digman 1990; McCrae and Costa 1997; Goldberg 1993), and it further assumes that individuals are informed, rational, variable, and proactive agents whose consistent dispositions manifest across situations (Seibert et al. 2021). Studies have confirmed that the five-factor (or dimension) personality model has predictive value in almost all behaviours related to personality (Wan et al. 2019). In other words, personality, when used as a scientific term, refers to the mental traits of individuals that characterize them in different situations and can therefore be used to predict their behaviour (Lavi et al. 2022). For example, personality has a psychological impact on how subjects perceive (Sindermann et al. 2020), interact (Hosťovecký and Prokop 2018), and use (Özbek et al. 2014) information technologies.

The five-factor personality structure has been widely replicated in different linguistic and cultural contexts (Kang 2022). The Big Five ABCD-M (Big Five ABCD-Minulescu Questionnaire) is the first personality questionnaire in the Romanian language; and being used in this research, it systematically follows the psycholexical approach of the psycholinguistic hypothesis regarding personality: the criteria according to which people spontaneously and coherently evaluate their own behaviour and that of their peers are specifically encoded in language (Minulescu 2008). This psycholexical tradition traces back to Allport & Odbert’s landmark work on trait adjectives (1936) and was popularized in the Big Five context by Goldberg (1993).

Although personality is a stable set of characteristics and tendencies that generate commonalities and differences among people with respect to their thoughts, feelings, and actions (Maddi 1989), the risk of smartphone addiction can configure pathological personality traits. (Popescu et al. 2022). Smartphone addiction is a vital problem within the global population with impairment that includes physical difficulties such as muscle pain and eye diseases, and psychological difficulties such as personality and psychiatric problems, auditory and tactile delirium (De-Sola et al. 2017; Lei et al. 2020), impaired sleep quality (Kheradmand et al. 2023), social anxiety (Popescu et al. 2022), depression, interpersonal relationship problems (Eichenberg et al. 2021), mood disorders, low self-esteem, and poor work performance (Tripathi 2018). More than smartphone addiction, in the literature, we even find that people can subjectively experience the smartphone as an extended part of themselves, feeling attachment to the device as well as the need to interact with it (Gertz et al. 2021).

The possibility of predicting smartphone addiction using the five personality factors as predictors has been studied in the literature over time, and the results have reflected the fact that extroverted people have a lower chance of becoming addicted to smartphones (Abd Rahim et al. 2020). Other studies have obtained exactly the opposite results, namely that the prevalence of Neuroticism and Extraversion in people with smartphone addiction is significantly higher than in those who are not addicted to smartphones (Eichenberg et al. 2021; Lei et al. 2020). Hussain et al. (2017) also found significant associations between problematic smartphone use and personality factors of Conscientiousness and Openness to Experience. In contrast, Ran (2022) reported no significant associations between high scores on Conscientiousness, Openness to Experience, or Agreeableness and the risk of smartphone addiction.

Complementary findings using alternative personality frameworks also support the link between personality and smartphone overuse. For instance, Kheradmand et al. (2023), using the Cloninger TCI (Temperament and Character Inventory) model, found that individuals prone to smartphone addiction scored significantly higher in novelty seeking, harm avoidance, and self-transcendence, and lower in persistence and self-directedness—a pattern that may indicate difficulties with self-regulation and goal-directed behaviour.

More recent studies have continued to investigate this association between personality factors and smartphone addiction. For example, as previously noted, Bhayangkara et al. (2024) found that Extraversion and Conscientiousness significantly predict smartphone addiction among university students, offering further empirical support for the predictive role of personality factors. Similarly, John et al. (2025) found that Neuroticism was positively associated with smartphone addiction while Agreeableness and Conscientiousness acted as significant protective factors. In a comparable line of research, Osorio et al. (2024) used a machine learning model based on the Big Five traits in a sample of teenagers, identifying Neuroticism and Conscientiousness as significant predictors of smartphone addiction.

## 3. Moderate Mediation Between Personality and Smartphone Technology Use

Although the relationship between personality and smartphone addiction, as shown above, has been studied in the literature, recent studies involving the Romanian population have not been found; moreover, there is no analysis of the relationship mentioned above, mediated by professional status and moderated by the cognitive-emotional coping, in the context of the ongoing digital transformation.

Coping is often defined as efforts to prevent or diminish threat, harm, and loss, or to reduce associated distress (Carver and Connor-Smith 2010). According to the cognitive behavioural theory, personality can influence an individual’s coping styles, and behaviours can lead to different levels of resilience (Lai and Li 2022). On the other hand, in Moodie et al. (2020), it is reported that brain activation patterns showed significant interactions between intensity and strategy (coping), suggesting that the brain engages strategies using distinct regulatory architectures with comparable behavioural efficacy and that these systems are differentially engaged depending on emotional intensity. More specifically, in Buhle et al. (2014), we find that reappraisal (as an adaptive coping strategy) is often used to reduce emotion and, consequently, it is associated with decreased self-reported negative affect ratings and activity in emotion-generating brain regions such as the amygdala. These findings indicate that coping strategies can moderate the effects of personality traits on the emotional reactions associated with these traits (for example, including but not limited to frustration, suspicion, anger, empathy, and affection—Agreeableness factor; satisfaction and guilt—Conscientiousness factor; joy and enthusiasm—Extraversion factor, etc.). Additionally, Su et al. (2024) demonstrated that psychological resilience moderates the relationship between negative affect and smartphone addiction among over 5000 students, emphasizing the protective role of adaptive coping mechanisms. In a similar vein, Cheng et al. (2024) highlighted that daily emotional experiences, particularly negative affect, predicted smartphone addiction through stress as a mediating factor, further illustrating the centrality of emotion regulation in the development of problematic phone use.

Compulsive smartphone use is considered a form of human–machine addiction characterized by a behavioural pattern similar to internet addiction or substance addiction (Ting and Chen 2020). For a clearer understanding of substance addiction, from which we drew an analogy to identify smartphone addiction, a possible definition can be found in the work of Koob and Le Moal (1997), where it is specified that addiction can be defined as a chronic, relapsing disorder, which was characterized by (i) a compulsion to seek and consume drugs, (ii) loss of control over drug use, and (iii) the appearance of a negative emotional state (e.g., dysphoria, anxiety, and irritability) that defines a motivational withdrawal syndrome when access to the drug is prevented. Therefore, it can be noticed that emotion is an integrative part of the addictive behaviour which, in its turn, triggers coping strategies, as presented above. Moreover, recent findings by Niskier et al. (2024) emphasize that behavioural habits tied to daily routines (e.g., screen use during meals and skipping extracurriculars) are significantly associated with problematic internet use in adolescents, reinforcing the idea that environmental and professional-contextual factors—such as occupational demands—may function as meaningful mediators in the escalation toward technology overuse. In conclusion, we can say that in the relationship between personality factors and mobile phone/smartphone addiction, adaptive coping acts with a moderating effect, the common element being emotion.

The relationship between personality factors and mobile phone addiction moderated by adaptive cognitive-emotional coping strategies will be investigated in this paper according to the current context of professional activities, as we showed at the beginning of this paper, that is, taking into account the requirements and demands of the “digital revolution” to which the population responds from the point of view of professional activity. In other words, the previously mentioned relationship will be analyzed in the context in which the mediator is represented by the professional status.

## 4. Objectives and Hypotheses of the Current Research

Taking into account the elements presented above, we propose in this research paper to evaluate, on a sample of adult Romanian citizens, how the main personality dimensions predict mobile phone addiction, both as a stand-alone relationship and as a relationship mediated by professional status and moderated by cognitive-emotional adaptive coping strategies. In other words, the purpose of the paper is to explain how cognitive-emotional adaptive coping strategies intervene in the context in which professional activity, in particular, exacerbates the relationship between personality and mobile phone use (even reaching addiction), and in the absence of these strategies to “sound an alarm” and to consider the development of educational and psychological counselling programmes by specialized organizations and decision makers, thus supporting the population.

Drawing on these aims, the study articulates the following objectives and hypotheses:

- The objectives of the current research are the following:
  - Identifying the relationships between personality factors and smartphone technology addiction;
  - Establishing the mediating role of professional status in the relationship moderated by adaptive cognitive-emotional coping strategies between personality and digitalization (represented by the smartphone).
- The current research hypotheses are the following:
  - H1: Personality factors can significantly predict smartphone addiction:
    H1.1: Extraversion will positively predict smartphone addiction.
    H1.2: Maturity will negatively predict smartphone addiction.
    H1.3: Agreeableness will positively predict smartphone addiction.
    H1.4: Conscientiousness will negatively predict smartphone addiction.
    H1.5: Self-actualization will positively predict smartphone addiction.
  - H2: Professional status mediates the relationship between personality (represented by the personality factors):
    H2.1: Extraversion (positive mediation-direct effect);
    H2.2: Maturity (negative mediation-indirect effect);
    H2.3: Agreeableness (positive mediation-direct effect);
    H2.4: Conscientiousness (negative mediation-indirect effect);
    H2.5: Self-actualization (positive mediation-direct effect) and
    digital transformation (represented by smartphone addiction).
  - H3: Professional status mediates the relationship between personality (represented by the personality factors):
    H3.1: Extraversion (positive mediation-direct effect);
    H3.2: Maturity (negative mediation-indirect effect);
    H3.3: Agreeableness (positive mediation-direct effect);
    H3.4: Conscientiousness (negative mediation-indirect effect);
    H3.5: Self-actualization (positive mediation-direct effect) and
    digital transformation (represented by smartphone addiction), moderate relationship (in the sense of reduction) to adaptive cognitive-emotional coping strategies.

In brief, H1 spells out the expected direct personality → addiction links; H2 specifies which of those links operate via professional status; H3 then tests how adaptive coping moderates those mediated effects.

**Figure 1:**
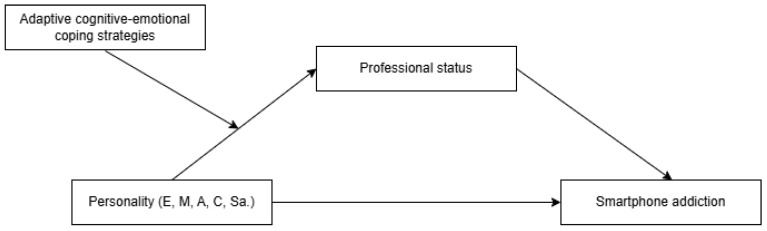
The hypothesized model. Note: E—Extraversion, M—Maturity, A—Agreeability, C—Conscientiousness, and Sa.—Self-actualization. Adaptive cognitive-emotional coping strategies: Acceptance, Positive Refocusing, Planning Refocusing, Positive Reappraisal, and Perspective-Taking.

In Figure 1, we can see the proposed theoretical model containing the three sets of hypotheses presented previously.

## 5. Method

### 5.1. Participants

The sample size was calculated a priori using the G*Power software (version 3.1.9.7). For a medium effect size, a statistical power of 0.80, a type I error of 0.05, and five predictors were needed; the minimum number of participants required was 92.

The final sample included 202 Romanian adults (48.8% men, 50.7% women, and 0.5% who did not declare their gender) aged between 18 and 61 years (M = 33.84, SD = 11.55). Participants represented a variety of professional statuses, including employees, freelancers, students, unemployed individuals, retirees, and homemakers. Most participants resided in urban areas (85.2%), while 14.8% lived in rural areas. Additionally, 59.4% of participants reported not having children.

### 5.2. Instruments

(a) The Big Five ABCD-M personality questionnaire

The personality factors were assessed using the Big Five ABCD-M questionnaire (Minulescu 2008), which was developed specifically for evaluating normal personality in Romanian adults. The instrument comprises 150 items distributed across five scales and 25 facets, with responses recorded on a 5-point Likert scale (ranging from 0 = total disagree to 4 = total agree). The five personality scales are represented by the following:

- Extraversion, with facets of Activism, Optimism, Humour, Interpersonal Ability, and Personal Affirmation;
- Maturity, encompassing Respect, Adaptation, Friendship, Inhibition Strength, and Ego Strength;
- Agreeableness, covering Altruism, Romanticism, Affective Warmth, Empathy, and Honesty;
- Conscientiousness, including Will/Perseverance, Spirit of Perfection, Rationality, Planning, and Self-discipline;
- Self-actualization, a distinctive scale (absent from many classical inventories) measuring Deepening, Tolerance, Refinement, Independence, and Creativity.

By integrating Maturity and Self-actualization alongside the core Big Five, the ABCD-M offers a richer portrait of personality as experienced in the Romanian cultural context.

In the current study, the 5 personality scales had very good internal consistency, with Cronbach’s alpha values of 0.928 (Extraversion), 0.954 (Maturity), 0.923 (Agreeableness), 0.933 (Conscientiousness), and 0.878 (Self-actualization). McDonald’s omega coefficients were similarly high with 0.936 (Extraversion), 0.956 (Maturity), 0.926 (Agreeableness), 0.935 (Conscientiousness), and 0.886 (Self-actualization), further supporting the reliability of the scales.

Note that while Extraversion, Agreeableness, and Conscientiousness align directly with their classical Big Five counterparts, the Maturity scale captures emotional stability akin to low Neuroticism, and the Self-actualization scale extends aspects of Openness to Experience by assessing personal growth, creativity, and independence beyond the traditional five-factor framework.

(b) Cognitive Emotion Regulation Questionnaire (CERQ)

The assessment of the adaptive cognitive-emotional coping strategies was carried out using the CERQ, adapted to the Romanian population. According to its manual (Garnefski et al. 2010), the CERQ is a multidimensional questionnaire, consisting of 36 items, designed to identify the cognitive coping strategies used following the experience of negative events or situations and can be used for both normal and clinical populations aged 12 years or older. The questionnaire assesses the following coping strategies: Acceptance, Positive Refocusing, Planning Refocusing, Positive Reappraisal, Perspective-Taking, Self-Blaming, Rumination, Catastrophizing, and Blaming Others. Out of these strategies, the first five, the adaptive ones, were used in the current research. Responses to the CERQ are recorded on a 5-point Likert scale (from 1—(almost) never to 5—(almost) always).

In the current research, the five cognitive-emotional coping scales demonstrated good internal consistency, with Cronbach’s alpha values of 0.854 (Acceptance), 0.851 (Positive Refocusing), 0.799 (Planning Refocusing), 0.833 (Positive Reappraisal), and 0.852 (Perspective-Taking). McDonald’s omega coefficients also indicated good reliability across all five adaptive coping scales—0.860, 0.853, 0.812, 0.835, and 0.859, respectively—further confirming the internal consistency of the CERQ subscales.

(c) Smartphone Addiction Scale—Short Version (SAS-SV)

The SAS-SV, Romanian version, was the third tool used in this research.

According to Tudor (2023), SAS-SV includes ten items to which a subject can respond on a Likert-type scale with six verbal anchors distributed gradually from 1—strong disagreement to 6—strong agreement, and the content of the items sample dimensions such as the difficulties that excessive smartphone use creates in everyday life, positive anticipation, reduction in social relationships simultaneously with the intensification of activities carried out in virtual space, difficulties in managing time spent on the smartphone, increased tolerance to use, etc.

As far as the current research work is concerned, the SAS-SV tool had good internal consistency, with Cronbach’s alpha value of 0.894. McDonald’s omega coefficient was also high (0.897), further confirming the reliability of the scale.

(d) Professional status

Professional status was assessed using a single-item question (“What is your current professional status?”), with the following six response options: (1) Employed, (2) Homemaker, (3) Self-employed (freelancer), (4) Retired (pensioner), (5) Unemployed, and (6) Student (pupil). For inclusion in the statistical analyses, these categorical responses were coded as ordinal values corresponding to their original listing (Employed = 1, Homemaker = 2, Self-employed = 3, Retired = 4, Unemployed = 5, and Student = 6). This coding scheme allowed professional status to function as a mediator in the modelling frameworks. These six categories capture qualitatively different smartphone-use contexts:

- Employed individuals face constant employer-driven communications and deadlines;
- Homemakers typically use their phones for domestic coordination and social contacts;
- Self-employed (freelancers) manage their own client communications and schedules;
- Retired participants have more sporadic, leisure-oriented usage;
- Unemployed respondents show variable patterns driven by job-search or personal use;
- Students navigate academic deadlines, coursework reminders, and campus notifications.

This categorization ensures that each mediator level corresponds to a distinct work- or life-context in which personality traits translate into smartphone engagement.

### 5.3. Procedure

The study was approved by the Ethics Committee (Scientific Council of University Research and Creation from West University of Timișoara, Romania) in August 2024 and was conducted in accordance with the Declaration of Helsinki (World Medical Association). All participants were informed about the study and provided informed consent.

The current study used a cross-sectional survey design, and responses were collected between the 12 October 2024 and 9 November 2024. This current study, which is part of a larger research project titled “The role of cognitive-emotional coping strategies in the relationship between personality and the digitalization process”, was pre-registered on the Open Science Framework platform (objectives, main hypotheses, study design, data collection procedure, measured variables, and statistical analysis plan) at: https://osf.io/e9apx/?view_only=e62096bfa5d34131bbd32d314125313b (accessed on 12 October 2024).

The survey, accessed online, through the Google Forms platform, voluntarily and without rewards, was conducted in Romania and contained demographic questions (year of birth, gender, marital status, professional status, and residential area (rural/urban)) followed by the following questionnaires: CERQ, Big Five ABCD-M, and SAS-SV.

With a view to collecting responses, the survey was promoted online, on social media platforms, professional platforms, and mobile messaging platforms.

Two hundred and five people intended to participate in the survey, but three people refused to give their informed consent to participate, and their participation was automatically excluded from the study. Participants were informed about the objectives of the research and also that they could withdraw at any time during the study. Each questionnaire was preceded by specific instructions, and the response time was approximately 50 min for each participant. The participants’ responses were recorded in the database completely anonymously.

The conditions for excluding responses received from participants were the following: the participant’s refusal to give consent to participate in the study—consent to participate in the study being the first condition to be met, before the study questions are displayed on the screen, and abandoning the questionnaire started by not completing it in full.

### 5.4. Data Analysis

In order to carry out the entire statistical analysis, the Jamovi software product, version 2.3.28.0 (The Jamovi Project 2022) was used, with the following modules:

- Descriptives for calculating descriptive statistics;
- Reliability Analysis for calculating the internal consistencies of scales (Cronbach’s alpha values);
- Correlation Matrix for analyzing the correlations between variables;
- Principal Component Analysis for analyzing the common method bias;
- Linear Regression for multiple linear regression analyses;
- Medmod—GLM Mediation Model for moderated mediation and bootstrap analyses.

## 6. Results

### 6.1. Common Method Bias Analysis

The common method bias was performed by analyzing all responses to the items of the three questionnaires used in the current research (Big Five ABCD-M, CERQ and, SAS-SV). The analysis was performed using the Principal Component Analysis module in Jamovi. For the variance explained by the first factor (first component), a value of 31% of the total variance (<50%) was obtained, indicating that there was no significant risk of common method bias (Podsakoff et al. 2003).

In addition, the correlation matrix of this study showed that the highest inter-construct correlation was below 0.558 and none exceeded the threshold of 0.90 (Bagozzi et al. 1991) indicating that there is no serious problem of common method bias. Furthermore, in order to detect multicollinearity, the analysis shows that the Variance Inflation Factor (VIF) of all constructs is lower than the threshold of 5 (Hair et al. 2011; Kock 2017), with the highest having the value of 1.908—Self-actualization. Also, Tolerance is higher than 0.1 for all constructs. Therefore, multicollinearity is excluded as a significant source of the common method bias.

### 6.2. Preliminary Analysis

Descriptive statistics and correlations between study variables can be seen in Table 1. To assess the assumptions for parametric analyses, we examined the distribution of the study variables using Q–Q plots, skewness, and kurtosis values. All skewness and kurtosis values fell well within the accepted range of ±2 (George and Mallery 2010) indicating a good approximation to normality for all variables, thus justifying the use of parametric analyses. Therefore, the use of parametric tests such as Pearson correlations and multiple linear regression analyses is appropriate.

Analyzing Table 1, we can see that mobile phone use is correlated with personality factors: Maturity—supporting H1.2 (r = −0.448, *p* < 0.001), Extraversion (r = −0.172, *p* < 0.05), and Conscientiousness (r = −0.255, *p* < 0.001), but contrary to our expectations, Extraversion and Conscientiousness are negatively correlated (in hypotheses H1.1 and H1.4 we expected a positive prediction). Also, taking into account the moderating role of the adaptive coping strategies (Acceptance, Positive Refocusing, Planning Refocusing, Positive Reappraisal, and Perspective-Taking) in the relationship between personality factors and smartphone addiction (criterion), we mention that the only correlation relationship, and that a very weak one between the moderator and the criterion, as we can see in Table 1, is the Positive Reappraisal coping strategy—smartphone addiction (SAS-SV), r = −0.160, *p* < 0.05. As to the correlation between the moderator (adaptive coping strategies) and the predictor (personality factors), the correlation relationships are weak, except for the relationship Maturity—SAS-SV and smartphone addiction (r = −0.448, *p* < 0.001).

### 6.3. The Relationship Between Personality Factors and Smartphone Technology Addiction

With a view to testing the first hypothesis, H1 (and sub-hypotheses H1.1–H1.5), a multiple linear regression analysis was performed, with the five personality factors as predictors and smartphone addiction as a dependent variable.

In order to simplify the interpretation of the results, we used standardized z-scores of the predictors and the dependent variable.

Analyzing the multiple linear regression model in Table 2, we could notice that the five personality factors are responsible for 32.1% of the variance in smartphone addiction, and the regression equation being statistically significant, F (7, 194) = 13.111, *p* < 0.001. Among the five factors, only one is a significant predictor of smartphone addiction: Maturity (β = −0.433, 95%CI [−0.559, −0.307], *p* < 0.001). In the analysis, we have included two covariates (i.e., gender and age) to exclude them as explanatory alternatives for the effects identified, as they proved to be substantially related to problematic mobile phone use in the previous studies (Hao et al. 2023).

The above-mentioned results lead us to state that hypothesis H1 is only partially supported by the analyzed data (only hypothesis H1.2 was confirmed). More specifically, as the value of the Maturity factor increases by one unit, the value of the dependent variable, smartphone addiction, decreases, on average, by the magnitude of the coefficient (β = −0.433).

### 6.4. The Relationship Between Personality Factors and Smartphone Technology Addiction, Mediated by the Professional Status

In Table 3, we find the results of the professional status mediation which intervened in the relationship between personality factors (Extraversion, Maturity, Agreeableness, Conscientiousness, and Self-actualization) and mobile phone addiction. The parallel mediation analysis indicated that professional status has a mediating role in the relationships between the following:

- The personality factor Extraversion and mobile phone addiction (indirect effect = −0.077, *p* < 0.01, 95%CI [−0.134, −0.02]), the mediation being complete (the relationship is completely mediated by the professional status, that is the indirect effect explains 100% of the variability of the total effect);
- The personality factor Self-actualization and mobile phone addiction (direct effect = 0.05, *p* < 0.05, 95%CI [0.003, 0.098]), the mediation being complete (the relationship is completely mediated by the professional status, that is the indirect effect explains 100% of the variability of the total effect).

The rest of the relationships between personality factors (Maturity, Agreeableness, and Conscientiousness) and mobile phone addiction are not mediated by the professional status, that is the hypotheses H2.3, H2.4, and H2.5 are not confirmed. However, by analyzing Table 3, we can see that there is a very strong direct relationship between the personality factor Maturity and mobile phone addiction (both for the direct effect and for the total effect). The same result was also highlighted by the multiple linear regression.

**Table 3:** Results of the mediation analysis.

				95% C.I. (a)		
Type	Effect	Estimate (β)	SE	Lower	Upper	*p*
Indirect	**Extraversion ⇒ Professional Status ⇒ SAS-SV**	**−0.077**	**0.029**	**−0.134**	**−0.02**	**0.008**
	Maturity ⇒ Professional Status ⇒ SAS-SV	−0.028	0.017	−0.061	0.005	0.095
	Agreeableness ⇒ Professional Status ⇒ SAS-SV	−0.034	0.02	−0.072	0.005	0.088
	Conscientiousness ⇒ Professional Status ⇒ SAS-SV	−0.004	0.019	−0.041	0.034	0.851
	**Self-actualization ⇒ Professional Status ⇒ SAS-SV**	**0.05**	**0.024**	**0.003**	**0.098**	**0.036**
Direct	Extraversion ⇒ SAS-SV	0	0.077	−0.151	0.152	0.996
	**Maturity ⇒ SAS-SV**	**−0.378**	**0.063**	**−0.501**	**−0.255**	**<0.001**
	Agreeableness ⇒ SAS-SV	0.145	0.073	0.002	0.288	0.048
	Conscientiousness ⇒ SAS-SV	−0.139	0.081	−0.298	0.021	0.089
	Self-actualization ⇒ SAS-SV	−0.088	0.081	−0.248	0.072	0.28
Total	Extraversion ⇒ SAS-SV	−0.077	0.076	−0.225	0.072	0.311
	**Maturity ⇒ SAS-SV**	**−0.406**	**0.064**	**−0.532**	**−0.281**	**<0.001**
	Agreeableness ⇒ SAS-SV	0.111	0.075	−0.035	0.257	0.136
	Conscientiousness ⇒ SAS-SV	−0.142	0.084	−0.307	0.022	0.09
	Self-actualization ⇒ SAS-SV	−0.038	0.082	−0.199	0.124	0.649

Note. All paths represent standardized estimates obtained after z-scoring the variables. Values in bold denote statistically significant findings (*p* < 0.05, *p* < 0.01, or *p* < 0.001). Abbreviations: C.I. = Confidence Interval; β = Standardized coefficient (Beta); SE = Standard Error; *p* = *p*-value (statistical significance).

In order to assess the significance of the multiple mediation model, we applied the bootstrap resampling technique with 5000 bootstrap replications identifying as significant relationships whose 95% confidence interval does not contain the value 0 (Liu et al. 2023; Yue et al. 2022; Kim et al. 2024). The results obtained from applying the bootstrap technique can be seen in Table 4.

In Table 4, we can see that the professional status remains as a mediator of the Extraversion/Self-actualization and smartphone addiction relationships, the 95%CI intervals for both relationships do not contain the value 0. The same situation is repeated for the direct and total effect between Maturity and smartphone addiction, the relationships remaining significant even after applying the bootstrap technique.

### 6.5. Personality Factors as Predictors of Smartphone Technology Use and Moderated Mediation

With a view to evaluating the last set of hypotheses, H3, a moderated mediation model was created, more precisely, evaluating the effects of the relationship between personality factors (Extraversion, Maturity, Agreeableness, Conscientiousness, and Self-actualization) and mobile phone addiction, moderated by adaptive cognitive-emotional coping (Acceptance, Positive Refocusing, Planning Refocusing, Positive Reappraisal, and Perspective-Taking) and mediated by the professional status. In Table 5 we can see the results of the analysis of this relationship and the analysis for various moderator levels can be found in the Supplementary Materials.

Hypothesis H3.1 (The professional status positively mediates the relationship between personality represented by the Extraversion factor and digital transformation (represented by smartphone addiction), and relationship moderated (in the sense of reduction) by the adaptive cognitive-emotional coping strategies) tested significant values only for the moderators:

- Positive refocusing (β = 0.162, 95%CI [0.007, 0.302], *p* < 0.05). Conditional indirect effect is significant only at low (−1 SD: β = −0.103, 95%CI [−0.176, −0.03], *p* < 0.05) and mean (0 SD: β = −0.069, 95%CI [−0.123, −0.016], *p* < 0.05) levels of the moderator among participants with a lower level of Extraversion.

A 5000-replicate percentile bootstrap yielded conditional indirect effects of β = −0.104, 95% CI [−0.181, −0.042], *p* = 0.004 at −1 SD; β = −0.070, 95% CI [−0.128, −0.025], *p* = 0.194 at 0 SD (mean); β = −0.036, 95% CI [−0.097, 0.014], *p* = 0.194 at +1 SD confirming that the moderated mediation pattern is robust at low coping levels, attenuates at the mean, and becomes non-significant at high coping levels.

- Positive reappraisal (β = 0.182, 95%CI [0.03, 0.282], *p* < 0.05). In this case, conditional indirect effect is significant also, only at low (−1 SD: β = −0.1, 95%CI [−0.173, −0.027], *p* < 0.01) and mean (0 SD: β = −0.066, 95%CI [−0.118, −0.014], *p* < 0.05) levels of the moderator and with a lower level of the Extraversion personality factor.

The 5000-replicate percentile bootstrap yielded conditional indirect effects of β = −0.098 (95% CI [−0.191, −0.039], *p* < 0.05) at −1 SD; β = −0.065 (95% CI [−0.120, −0.023], *p* < 0.01) at 0 SD (mean); β = −0.031 (95% CI [−0.077, 0.015], *p* = 0.176) at +1 SD confirming that the moderated mediation model is robust at low coping levels, attenuates at the mean, and becomes non-significant at high coping levels.

Hypothesis H3.2 (The professional status negatively mediates the relationship between personality represented by the Maturity factor and digital transformation (represented by smartphone addiction), relationship moderated (in the sense of reduction) by the adaptive cognitive-emotional coping strategies) tested significant values only for the moderators:

- Acceptance (β = −0.166, 95%CI [−0.35, −0.037], *p* < 0.05). Conditional indirect effect is significant only at the high (+1 SD: β = −0.068, 95%CI [−0.126, −0.01], *p* < 0.05) level of the moderator among participants with lower Maturity.

Using a 5000-replicate percentile bootstrap, the conditional indirect effects were estimated as β = −0.011, 95% CI [−0.021, 0.053], *p* = 0.524 at −1 SD; β = −0.020, 95% CI [−0.059, 0.004], *p* = 0.219 at 0 SD (mean); β = −0.052, 95% CI [−0.129, −0.007], *p* = 0.094 at +1 SD indicating that the indirect effect is non-significant at low and mean levels of the moderator but becomes significant at high levels, demonstrating the robustness of the moderated mediation model. According to Hayes (2013), the bootstrap confidence interval is the benchmark for establishing mediation significance; as it excludes zero only at +1 SD, this confirms that the moderated mediation effect is statistically robust under high levels of the moderator.

- Perspective-Taking (β = −0.145, 95%CI [−0.274, −0.015], *p* < 0.05). In this case, conditional indirect effect is significant also, only at the high (+1 SD: β = −0.055, 95% CI [−0.103, −0.006], *p* < 0.05) level of the moderator among participants with lower levels of the Maturity factor.

A 5000-replicate percentile bootstrap analysis revealed conditional indirect effects of β = 0.007, 95% CI [−0.044, 0.052], *p* = 0.752 at −1 SD; β = −0.021, 95% CI [−0.063, 0.006], *p* = 0.23 at 0 SD (mean); β = −0.050, 95% CI [−0.113, −0.006], *p* = 0.063 at +1 SD indicating that the indirect effect is non-significant at low and mean levels of the moderator but becomes significant at high levels (+1 SD CI excludes zero), demonstrating the robustness of the moderated mediation model under high coping levels.

Hypothesis H3.3 (The professional status positively mediates the relationship between personality represented by the Agreeableness factor and digital transformation (represented by smartphone addiction), relationship moderated (in the sense of reduction) by the adaptive cognitive-emotional coping strategies) tested significant values only for the moderators:

- Acceptance (β = 0.15, 95%CI [0.006, 0.272], *p* < 0.05). Conditional indirect effect was significant only at the low (−1 SD: β = −0.061, 95% CI [−0.115, −0.006], *p* < 0.05) level of the moderator and with a lower level of the Agreeableness personality factor.

The 5000-replicate percentile bootstrap yielded conditional indirect effects of β = −0.046, 95% CI [−0.177, −0.010], *p* = 0.057 at −1 SD; β = −0.023, 95% CI [−0.114, 0.007], *p* = 0.19 at 0 SD (mean); β = −0.0006, 95% CI [−0.0792, 0.073], *p* = 0.976 at +1 SD indicating that the moderated mediation effect is also significant only at low levels of Acceptance (−1 SD CI excludes zero), not at the mean or high levels.

- Planning Refocusing (β = 0.184, 95%CI [0.015, 0.3], *p* < 0.05). In this case, conditional indirect effect was also significant only at the low (−1 SD: β = −0.064, 95% CI [−0.12, −0.007], *p* < 0.05) level of the moderator and at lower levels of Agreeableness.

Also, the 5000-replicate percentile bootstrap yielded conditional indirect effects of β = −0.061, 95% CI [−0.130, −0.007], *p* = 0.052 at −1 SD; β = −0.028, 95% CI [−0.078, 0.005], *p* = 0.177 at 0 SD (mean); β = 0.004, 95% CI [−0.053, 0.055], *p* = 0.879 at +1 SD confirming that the moderated mediation effect is significant only at low levels of Planning Refocusing (−1 SD CI excludes zero), not at the mean or high levels.

Hypothesis H3.4 (The professional status positively mediates the relationship between personality represented by the Conscientiousness factor and digital transformation (represented by smartphone addiction), relationship moderated (in the sense of reduction) by the adaptive cognitive-emotional coping strategies) did not produce any significant moderated mediation effects.

Hypothesis H3.5 (The professional status positively mediates the relationship between personality represented by the Self-actualization factor and digital transformation (represented by smartphone addiction), relationship moderated (in the sense of reduction) by adaptive cognitive-emotional coping strategies) also showed no evidence of moderated mediation under any of the coping strategies tested.

Full 5000-replicate bootstrap results for all direct, indirect, and total effects in the moderated mediation analyses are provided in the Supplementary Materials.

## 7. Discussion

The aim of the current study was to analyze the relationships between personality factors from the Big Five ABCD-M model and smartphone addiction in a sample of adult Romanian citizens, both as a direct relationship and by first taking into account the mediator professional status and finally integrating the moderator adaptive cognitive-emotional coping strategies into the relationship.

The descriptive statistical analysis showed that respondents recorded significant negative levels of Maturity (r = −0.448, *p* < 0.001) and Conscientiousness (r = −0.255, *p* < 0.001) and a low level of Extraversion (r = −0.172, *p* < 0.05) in relation to smartphone addiction. As to the adaptive coping, only one significant correlation was found with Positive Reappraisal (r = −0.16, *p* < 0.05), but with a low and negative effect in relation to smartphone addiction.

The first set of hypotheses, H1 (H1.1–H1.5) aimed to test the relationships that form between personality factors and smartphone addiction, and the results highlighted the fact that the personality factor Maturity is significantly associated with mobile phone addiction (β = −0.433, *p* < 0.001). As we can see, the relationship between the Maturity factor and smartphone addiction is of medium and reversed strength. This result indicates that people with a higher level of Maturity tend to be less addicted to smartphones. The findings by Su et al. (2024) align with this interpretation, showing that psychological resilience—often associated with mature emotional regulation—buffers the emotional impact of smartphone addiction by moderating its association with negative affect. This suggests that stable personality resources, such as emotional maturity and resilience, may play a protective role in managing the emotional consequences of compulsive digital behaviour. As to Extraversion, Agreeableness or Self-actualization, we would have expected them to correlate positively with smartphone addiction given the characteristics of these personality factors regarding interpersonal relationships and Openness to Experience. The lack of these relationships may indicate the fact that not the personality traits in particular are those that generate an individual’s tendency to compulsively use their mobile phone, but other psycho-emotional elements that will need to be investigated in future research studies. The lack of a relationship between Conscientiousness and smartphone addiction may be explained by the rationality, planning, and self-discipline elements characteristic of this factor.

The second set of hypotheses tested, H2 (H2.1–H2.5), referred to the relationship between personality and smartphone addiction, a relationship moderated by the professional status. As we can see in Table 3, the personality factor Maturity retained its quality as a predictor, excluding any mediation regarding smartphone addiction, the direct relationship and the total relationship remaining significant and reversed, as we identified in the first set of hypotheses, H1. These relationships remain significant even after applying the bootstrap technique. This result is, however, consistent with what we find in the literature and also with our expectations: adults, over 30 years of age, present a lower risk of behavioural addiction due to increased emotional and self-regulation skills, for example, using the phone less for socializing (van Deursen et al. 2015) or just being less interested in technology (Charness and Bosman 1992), compared to adolescents or young adults, who have a higher probability of smartphone addiction, given their predisposition for excessive use of new technologies (Alan and Guzel 2020).

Other results obtained from the analysis performed on testing the second set of hypotheses, H2, were related to the fact that the relationship between the personality factor Extraversion and mobile phone addiction is completely mediated by the professional status, the relationship being negative, indirect, and invalidating hypothesis H2.1, which refers to a positive relationship. In other words, the professional status, a contextual element, fully explains the influence of the Extraversion factor on mobile phone addiction. A similar mechanism is reflected in the findings by Cheng et al. (2024), who demonstrated that daily emotional experiences—especially negative affect—contribute to smartphone addiction through the mediating role of stress. Their results underscore the importance of affective and psychological mediators, such as stress, in understanding how contextual or situational variables influence compulsive smartphone use. Since the indirect effect has the value −0.077, *p* < 0.01, 95%CI [−0.134, −0.02], we can infer that, in general, more extroverted people tend to be less addicted to mobile phones, but addiction varies depending on the role of the professional status. The relationship between the Extraversion factor and mobile phone addiction is also found in the literature as either negligible (Horwood and Anglim 2018), or unlikely (Abd Rahim et al. 2020), or non-existent (Hussain et al. 2017; Ran 2022) given that extroverted individuals find alternative ways to satisfy their social needs, turning less to smartphones, compared to introverted individuals, who choose the opposite (Abd Rahim et al. 2020). However, the relationship between Extraversion and smartphone addiction is possible, as we have shown above, through the mediator professional status.

Another result obtained from the analysis carried out on the testing of the second set of hypotheses, H2, referred to the fact that the relationship between the personality factor Self-actualization and mobile phone addiction is completely mediated by the professional status, the relationship being positive, direct, and validating hypothesis H2.5. Therefore, from this result, we can understand that people with a higher level of the Self-actualization factor have a tendency to show a greater dependence on the mobile phone. In fact, the result obtained is not surprising; the more frequent use of the mobile phone is really justified in relation to professional status, taking into account the fact that the Self-actualization factor also includes the concern for personal evolution in relation to oneself as well as to the professional environment.

It is also necessary to mention that the professional status remains a mediator of the relationships between Extraversion/Self-actualization and smartphone addiction, meaning that the relationships are confirmed as significant even after applying the bootstrap technique, with 5000 replications.

The last set of hypotheses tested, H3, referred to the relationship between personality factors and mobile phone addiction, a relationship mediated by the professional status and moderated by the adaptive cognitive-emotional coping strategies, as we can see in Figure 1. The moderated mediation analysis, according to Table 5, highlighted the following results:

- The professional status positively mediates the relationship between personality represented by the Extraversion factor and smartphone addiction, a relationship moderated (in the sense of reduction) by adaptive cognitive-emotional coping strategies: Positive Refocusing and Positive Reappraisal.

The existence of a significant effect of the relationship between Extraversion and smartphone addiction, moderated by the professional status, has already been demonstrated following the analysis of the second set of hypotheses. The novelty brought by the third set of hypotheses is represented by the addition of the adaptive coping strategies as a moderator of the relationship between Extraversion and professional status, and the result was significant only for the coping strategies: Positive Refocusing and Positive Reappraisal, amplifying the relationship. Thus, we can conclude that the two adaptive coping strategies determine the individual to use the mobile phone more frequently in a professional context. These results are consistent with the literature, where we find associations between mobile phone addiction and adaptive cognitive-emotional coping (Hussain et al. 2023; Shahidin et al. 2022).

- The professional status negatively mediates the relationship between personality represented by the Maturity factor and smartphone addiction, a relationship moderated (in the sense of reduction) by the adaptive cognitive-emotional coping strategies: Acceptance and Perspective-Taking.

If in the case of the two previously tested sets of hypotheses, H1 and H2, the Maturity factor was shown to have a direct (not mediated) effect on smartphone addiction, the relationship being indirect, in the case of the third set of hypotheses, H3, the difference is made by the adaptive cognitive-emotional coping added to the relationship, and which allowed, in the case of Acceptance and Perspective-Taking strategies, the mediation of the relationship through the professional status. Therefore, we can say that people with a higher level of Maturity may use the mobile phone more frequently, in a professional context, once they also accept this professional attribution on the one hand, and on the other hand, with the aim of being constantly up to date with all aspects related to the workplace (even in their free time). The research studies admit that frequent use of the phone can be caused by purely practical reasons, such as work commitments (Linden et al. 2021), on the one hand. On the other hand, in the literature, we find studies that confirm the influence of positive coping strategies on smartphone addiction (Qiu et al. 2024).

- The professional status positively mediates the relationship between personality represented by the Agreeableness factor and smartphone addiction, a relationship moderated (in the sense of reduction) by the adaptive cognitive-emotional coping strategies: Acceptance and Planning Refocusing.

The personality factor Agreeableness in relation to mobile phone addiction, a significant relationship, appears for the first time following the analysis of the third set of hypotheses, H3, of this study, but the effect is significant only in the context in which the relationship is mediated by the professional status and moderated by the coping strategies Acceptance and Planning Refocusing. In other words, in the case of people characterized by affective warmth, empathy, honesty, and relationship capacity, the use of mobile phone, in a professional context, once the individual assumes his full involvement in all aspects related to the profession and/or is in search of solutions, depending on the impediments that have arisen in professional life, may increase. The simple, direct relationship between Agreeableness and smartphone addiction relationship is also indirectly confirmed by the literature, where we find that people with a high degree of agreeableness do not excessively use smartphones for social networks nor do they become addicted to these devices (Chang et al. 2022), a situation already confirmed following the analysis of the sets of hypotheses H1 and H2 within this study. At the same time, we find in the literature that the use of positive coping strategies can moderate the relationship between personality dimensions and smartphone addiction, by reducing the impact of traits that predispose to addiction, for example, in the context of task performance (Wei et al. 2024). Similarly, Qiu et al. (2024) showed that positive coping styles—acting as adaptive emotion regulation mechanisms—can reduce the negative impact of anxiety and intolerance of uncertainty on smartphone addiction, supporting the idea that adaptive coping moderates the vulnerability to problematic smartphone use.

More recent international evidence further supports and expands upon our results. For example, Wang and Ma (2024) demonstrated that social isolation contributes to smartphone addiction through the mediators of loneliness and COVID-19-related anxiety, underscoring the influence of situational stressors on excessive technology use. Tu et al. (2023) reported that stress intensity and specific smartphone-use motivations differ by gender, with stress more strongly predicting social-use addiction in females. Lastly, Yin et al. (2024) found that emotion regulation and self-control significantly mediated the relationship between family happiness and smartphone addiction among college students, reinforcing our conceptualization of adaptive coping as a protective buffer against addictive use patterns.

Although smartphone technology has been studied countless times to date, in the literature, we do not find an approach similar to the context proposed in this study: personality factors as predictors of smartphone addiction, moderated by positive coping strategies, taking into account the professional status. This approach, in addition to completing the literature from a theoretical–conceptual point of view, also brings valuable information due to the fact that the sample is made up of adult Romanian citizens (not students as we find in most existing studies) and samples with these characteristics have a very low percentage in the world of specialized literature. However, although smartphone technology has been intensively used for more than a decade in Romania (in 2015, 27% of Romanians owned a mobile phone and used it at least once a month, and in 2024 the estimated percentage reached 82% with a growing trend, forecasting a percentage of 84% for 2025 (Statista 2021)), the presence of this study can help, now perhaps more than ever, as we are in the midst of a digital “transformation”, on the one hand the population to raise its emotional awareness of the way in which smartphone devices are used, and on the other hand the companies, in order to optimize their employees’ activities, avoiding their overload by understanding perhaps more clearly the psycho-emotional implications resulting from overload.

## 8. Limitations and Future Research Directions

Although the current descriptive, exploratory, differential, and correlational study provides a series of valuable information, being the first conducted on this topic, on the Romanian population, it nevertheless has a series of limitations. The first limitation is related to the study design, which is cross-sectional, meaning that it excludes the inference of causal relationships between the variables examined in the study (Liu et al. 2023), therefore future research can validate the results of this study in longitudinal settings as well as by increasing the sample size (Kanwal and Isha 2022). Although our priori GPower analysis indicated a minimum N = 92 for a five-predictor regression, G*Power does not account for the additional complexity of our moderated-mediation GLMs. Future studies should employ Monte Carlo-based sample size planning (Donnelly et al. 2022) to ensure adequate power for both indirect and interaction effects.

In our mediation models, professional status was coded on an ordinal scale of six categories (Employed, Homemaker, Self-employed, Retired, Unemployed, and Student). We acknowledge that the exact ordering of these categories could be debated and that some groups (e.g., Homemaker, Retired, and Unemployed) had relatively few participants, which may limit power to detect mediating effects. As a sensitivity check, we also re-estimated the mediation models using dummy-coded indicators for all six professional status categories; despite unequal subsample sizes, each auxiliary model’s indirect effect mirrored the direction and significance of the original ordinal-status specification, confirming the robustness of our mediation findings. Future studies with larger and more balanced samples should explore alternative coding schemes—such as category collapsing or dummy-variable approaches—to further validate the mediator’s role.

The current study focused in particular on the five general personality factors (Extraversion, Maturity, Agreeableness, Conscientiousness, and Self-actualization), but future studies may also consider analyzing facets of factors related to smartphone addiction, in a professional context or in general, taking into account the influence of coping strategies. Also, the current study was limited to including only adaptive coping strategies in the analysis. Similarly, future studies may also include maladaptive coping strategies, and if an effect is identified, proposals for psychological intervention or even national programmes may be put forward.

Another limitation is related to the fact that the current study is based on self-reported perceptions of respondents, and this could generate subjective results (Kim et al. 2024).

We also consider it particularly important for the following research works which will relate to the use of smartphone technology to measures the level of stress, anxiety (Sarhan 2024), and depression (Nikolic et al. 2023) in the population within the future research samples, given the existence in the literature of the association between this type of emotional distress and mobile phone use.

Even though the current study is among the first of its kind, both in terms of the literature and the specifics of the sample, and it brings forward important information relating to the Romanian population, we nevertheless consider that future research should bring even more clarifications and more specific information focusing on all these previously mentioned elements.

## Figures and Tables

**Table 1:** Descriptive statistics and correlations between the investigated variables.

	Mean	SD	1	2	3	4	5	6	7	8	9	10	11	12	13	14
Acceptance [1]	3.344	1.041	—													
Positive Refocusing [2]	3.389	0.974	0.416 ***	—												
Planning Refocusing [3]	4.147	0.767	0.214 **	0.309 ***	—											
Positive Reappraisal [4]	4.198	0.788	0.273 ***	0.466 ***	0.541 ***	—										
Perspective-Taking [5]	3.64	0.953	0.305 ***	0.413 ***	0.32 ***	0.445 ***	—									
Extraversion [6]	2.866	0.574	0.042	0.3 ***	0.284 ***	0.355 ***	0.205 **	—								
Maturity [7]	3.204	0.695	−0.04	−0.138	0.043	0.177 *	−0.025	0.098	—							
Agreeableness [8]	2.748	0.571	0.285 ***	0.302 ***	0.321 ***	0.303 ***	0.353 ***	0.343 ***	0.039	—						
Conscientiousness [9]	2.9	0.573	0.058	0.204 **	0.295 ***	0.347 ***	0.205 **	0.53 ***	0.25 ***	0.456 ***	—					
Self-actualization [10]	2.953	0.46	0.146 *	0.32 ***	0.321 ***	0.407 ***	0.184 **	0.492 ***	0.078	0.521 ***	0.558 ***	—				
SAS-SV [11]	23.02	10.06	0.067	0.061	−0.049	−0.16 *	0.082	−0.172 *	−0.448 ***	−0.015	−0.255 ***	−0.128	—			
Professional status [12]			0.008	−0.091	−0.069	−0.25 ***	−0.092	−0.317 ***	−0.157 *	−0.169 *	−0.179 *	−0.042	0.279 ***	—		
Age (years) [13]	33.842	11.552	0.014	0.175 *	0.08	0.266 ***	0.07	0.155 *	0.183 **	0.163 *	0.189 **	0.121	−0.284 ***	−0.478 ***	—	
Gender (0/1/2) [14]			0.044	0.023	0.186 **	0.135	0.035	−0.001	0.235 ***	0.199 **	0.007	0.057	0.084	0.006	0.275 ***	—

Note: * *p* < 0.05, ** *p* < 0.01, *** *p* < 0.001, SD = Standard Deviation, 1 = Acceptance, 2 = Positive Refocusing, 3 = Planning Refocusing, 4 = Positive Reappraisal, 5 = Perspective-Taking, 6 = Extraversion, 7 = Maturity, 8 = Agreeableness, 9 = Conscientiousness, 10 = Self-actualization, 11 = SAS-SV, 12 = Professional status, 13 = Age (years), and 14 = Gender (0 = M, 1 = F, 2 = N/A).

**Table 2:** The regression analysis to test the relationship between personality factors and smartphone addiction.

Predictor	β	SE	*p*
Intercept		0.359	0.335
Extraversion	−0.049	0.073	0.501
Maturity	−0.433	0.064	<0.001
Agreeableness	0.072	0.129	0.328
Conscientiousness	−0.081	0.081	0.319
Self-actualization	−0.046	0.079	0.565
Age (years)	−0.256	0.063	<0.001
Gender	0.245	0.065	<0.001

R^2^ = 0.321, *p* < 0.001.

**Table 4:** The results following the application of the bootstrap technique.

				95% C.I. (a)		
**Type**	Effect	Estimate (β)	SE	Lower	Upper	*p*
Indirect	**Extraversion ⇒ Professional Status ⇒ SAS-SV**	**−0.077**	**0.027**	**−0.133**	**−0.028**	**0.004**
	**Self-actualization ⇒ Professional Status ⇒ SAS-SV**	**0.05**	**0.025**	**0.011**	**0.108**	**0.045**
Direct	**Maturity ⇒ SAS-SV**	**−0.378**	**0.077**	**−0.523**	**−0.221**	**<0.001**
Total	**Maturity ⇒ SAS-SV**	**−0.406**	**0.075**	**−0.554**	**−0.26**	**<0.001**

Note. All paths represent standardized estimates obtained after z-scoring the variables. Values in bold denote statistically significant findings (*p* < 0.05, *p* < 0.01, or *p* < 0.001). Abbreviations: C.I. = Confidence Interval; β = Standardized coefficient (Beta); SE = Standard Error; *p* = *p*-value (statistical significance).

**Table 5:** The results of the moderation analysis.

Moderator	Interaction	Estimate (B)	SE	Lower	Upper	β	*p*
Acceptance	Extraversion: Acceptance ⇒ Professional Status	0.097	0.073	−0.045	0.24	0.102	0.181
	**Maturity: Acceptance ⇒ Professional Status**	**−0.194**	**0.08**	**−0.35**	**−0.037**	**−0.166**	**0.015**
	**Agreeableness: Acceptance ⇒ Professional Status**	**0.139**	**0.068**	**0.006**	**0.272**	**0.15**	**0.04**
	Conscientiousness: Acceptance ⇒ Professional Status	−0.133	0.088	−0.306	0.04	−0.128	0.132
	Self-actualization: Acceptance ⇒ Professional Status	0.048	0.075	−0.099	0.196	0.051	0.52
Positive Refocusing	**Extraversion: Positive Refocusing ⇒ Professional Status**	**0.154**	**0.075**	**0.007**	**0.302**	**0.162**	**0.04**
	Maturity: Positive Refocusing ⇒ Professional Status	−0.054	0.071	−0.193	0.086	−0.051	0.449
	Agreeableness: Positive Refocusing ⇒ Professional Status	0.049	0.081	−0.11	0.207	0.05	0.548
	Conscientiousness: Positive Refocusing ⇒ Professional Status	−0.044	0.088	−0.216	0.128	−0.045	0.619
	Self-actualization: Positive Refocusing ⇒ Professional Status	−0.015	0.089	−0.19	0.159	−0.015	0.862
Planning Refocusing	Extraversion: Planning Refocusing ⇒ Professional Status	−0.066	0.071	−0.204	0.073	−0.073	0.352
	Maturity: Planning Refocusing ⇒ Professional Status	0.057	0.071	−0.082	0.195	0.058	0.425
	**Agreeableness: Planning Refocusing ⇒ Professional Status**	**0.157**	**0.073**	**0.015**	**0.3**	**0.184**	**0.03**
	Conscientiousness: Planning Refocusing ⇒ Professional Status	0.032	0.102	−0.167	0.232	0.033	0.75
	Self-actualization: Planning Refocusing ⇒ Professional Status	−0.138	0.084	−0.303	0.026	−0.176	0.099
Positive Reappraisal	**Extraversion: Positive Reappraisal ⇒** Professional Status	**0.156**	**0.064**	**0.03**	**0.282**	**0.182**	**0.015**
	Maturity: Positive Reappraisal ⇒ Professional Status	−0.089	0.061	−0.208	0.03	−0.102	0.144
	Agreeableness: Positive Reappraisal ⇒ Professional Status	0.038	0.081	−0.121	0.197	0.042	0.639
	Conscientiousness: Positive Reappraisal ⇒ Professional Status	−0.039	0.091	−0.218	0.14	−0.04	0.67
	Self-actualization: Positive Reappraisal ⇒ Professional Status	−0.062	0.071	−0.202	0.077	−0.08	0.382
Perspective-Taking	Extraversion: Perspective-Taking ⇒ Professional Status	0.1	0.071	−0.039	0.239	0.104	0.158
	**Maturity: Perspective-Taking ⇒ Professional Status**	**−0.144**	**0.066**	**−0.274**	**−0.015**	**−0.145**	**0.029**
	Agreeableness: Perspective-Taking ⇒ Professional Status	−0.003	0.077	−0.155	0.148	−0.003	0.964
	Conscientiousness: Perspective-Taking ⇒ Professional Status	0.016	0.088	−0.155	0.188	0.016	0.852
	Self-actualization: Perspective-Taking ⇒ Professional Status	−0.018	0.08	−0.175	0.138	−0.019	0.82

Note. All paths represent standardized estimates obtained after z-scoring the variables. Values in bold denote statistically significant findings (*p* < 0.05, *p* < 0.01, or *p* < 0.001). Abbreviations: C.I. = Confidence Interval; Estimate (B) = Unstandardized Regression Coefficient (B); β = Standardized coefficient (Beta); SE = Standard Error; *p* = *p*-value (statistical significance).

## Data Availability

The original contributions presented in the study are included in the article, further inquiries can be directed to the corresponding author.

## Supplementary Materials

The following supporting information can be downloaded at: https://www.mdpi.com/article/10.3390/jintelligence13070086/s1.

## Abbreviations

The following abbreviations are used in this manuscript:

Big Five ABCD-M	Big Five ABCD-Minulescu Questionnaire
β	Beta (Standardized coefficient)
CERQ	Cognitive Emotion Regulation Questionnaire
CI	Confidence Interval
COVID-19	Coronavirus Disease 2019
Estimate (B)	Unstandardized Regression Coefficient (B)
F	Function
GLM	General Linear Model
H	Hypothesis
M	Mean
*p*	*p*-value (statistical significance)
Personality (E, M, A, C, Sa.)	Personality (Extraversion, Maturity, Agreeability, Conscientiousness, and Self-actualization)
Q–Q plot	Quantile–Quantile plot
r	Pearson correlation coefficient
R^2^	Coefficient of Determination
SAS-SV	Smartphone Addiction Scale—Short Version
SD	Standard Deviation
SE	Standard Error
TCI	Temperament and Character Inventory
VIF	Variance Inflation Factor

## References

[B1-jintelligence-13-00086] Abd Rahim Nurul Ain, Siah Yih Huang, Tee Xiang Yi, Siah Poh Chua (2020). Smartphone Addiction: Its Relationships to Personality Traits and Types of Smartphone Use. International Journal of Technology in Education and Science.

[B2-jintelligence-13-00086] Akbari Mehdi, Seydavi Mohammad, Jamshidi Shiva, Marino Claudia, Spada Marcantonio M. (2023). The Big-five personality traits and their link to problematic and compensatory Facebook use: A systematic review and meta-analysis. Addictive Behaviors.

[B30-jintelligence-13-00086] Alan Rana, Guzel Halime Senay (2020). The ınvestigation of the relationship between smartphone addiction, and problem-solving skills and ways of coping with stress. Dusunen Adam: The Journal of Psychiatry and Neurological Sciences.

[B3-jintelligence-13-00086] Allport G. W., Odbert H. S. (1936). Trait-names: A psycho-lexical study. Psychological Monographs.

[B4-jintelligence-13-00086] American Psychiatric Association (2024). American Adults Express Increasing Anxiousness in Annual Poll; Stress and Sleep Are Key Factors Impacting Mental Health. https://www.psychiatry.org/news-room/news-releases/annual-poll-adults-express-increasing-anxiousness.

[B5-jintelligence-13-00086] APA (2023). Percentage of U.S. Adults That Were Anxious About Select Aspects of Their Lives in 2020, 2021, and 2023* [Graph]. Statista.

[B6-jintelligence-13-00086] AXA (2022). Prevalence of Anxiety, Depression, and Stress in Selected European Countries as of 2022 [Graph]. Statista.

[B7-jintelligence-13-00086] Bagozzi Richard P., Yi Youjae, Phillips Lynn W. (1991). Assessing Construct Validity in Organizational Research. Administrative Science Quarterly.

[B8-jintelligence-13-00086] Bano Shohar, Shah Ubaid Ullah, Ali Sabha (2019). Personality and Technology: Big Five Personality Traits as Descriptors of Universal Acceptance and Usage of Technology UTAUT. Library Philosophy and Practice (e-journal).

[B9-jintelligence-13-00086] Bhayangkara Noer Ichsan, Lerik Mariana Dinah Ch, Benu Juliana Marlin Y. (2024). Smartphone Addiction Reviewed from Big Five Personality in College Students. Journal of Health and Behavioral Science.

[B10-jintelligence-13-00086] Blake Holly, Hassard Juliet, Singh Jasmeet, Teoh Kevin (2024). Work-related smartphone use during off-job hours and work-life conflict: A scoping review. PLoS Digital Health.

[B11-jintelligence-13-00086] Buhle Jason T., Silvers Jennifer A., Wager Tor D., Lopez Richard, Onyemekwu Chukwudi, Kober Hedy, Weber Jochen, Ochsner Kevin N. (2014). Cognitive Reappraisal of Emotion: A Meta-Analysis of Human Neuroimaging Studies. Cerebral Cortex.

[B12-jintelligence-13-00086] Carver Charles S., Connor-Smith Jennifer (2010). Personality and Coping. Annual Review of Psychology.

[B13-jintelligence-13-00086] Chang Kuo, Li Xue, Zhang Lei, Zhang Hui (2022). A Double-Edged Impact of Social Smartphone Use on Smartphone Addiction: A Parallel Mediation Model. Frontiers in Psychology.

[B14-jintelligence-13-00086] Charness Neil, Bosman Elizabeth A., Craik Fergus I. M., Salthouse Timothy A. (1992). Human factors and age. The Handbook of Aging and Cognition.

[B15-jintelligence-13-00086] Cheng Qiuping, Zhou Ying, Zhu Hongying, Wang Qunlong, Peng Wei (2024). Relationships between daily emotional experiences and smartphone addiction among college students: Moderated mediating role of gender and mental health problems. Frontiers in Psychology.

[B16-jintelligence-13-00086] CivicScience (2022). Digital Device Addiction Among Users in the United States as of September 2022, by Stress Level [Graph]. Statista.

[B17-jintelligence-13-00086] De-Sola José, Talledo Hernán, Rubio Gabriel, de Fonseca Fernando Rodríguez (2017). Development of a Mobile Phone Addiction Craving Scale and Its Validation in a Spanish Adult Population. Frontiers in Psychiatry.

[B18-jintelligence-13-00086] Devi Khumukchan A., Singh Sudhakar K. (2023). The hazards of excessive screen time: Impacts on physical health, mental health, and overall well-being. Journal of Education and Health Promotion.

[B19-jintelligence-13-00086] Digman John M. (1990). Personality Structure: Emergence of the Five-Factor Model. Annual Review of Psychology.

[B20-jintelligence-13-00086] Donnelly Samuel, Jorgensen Terrence D., Rudolph Cort W. (2022). Power analysis for conditional indirect effects: A tutorial for conducting Monte Carlo simulations with categorical exogenous variables. Behavior Research Methods.

[B21-jintelligence-13-00086] Eichenberg Christiane, Schott Markus, Schroiff Athina (2021). Problematic Smartphone Use—Comparison of Students With and Without Problematic Smartphone Use in Light of Personality. Frontiers in Psychiatry.

[B22-jintelligence-13-00086] Elhai Jon D., Gallinari Elizabeth F., Rozgonjuk Dmitri, Yang Haibo (2020). Depression, anxiety and fear of missing out as correlates of social, non-social and problematic smartphone use. Addictive Behaviors.

[B23-jintelligence-13-00086] Elhai Jon D., Casale Silva, Montag Christian (2025). Worry and fear of missing out are associated with problematic smartphone and social media use severity. Journal of Affective Disorders.

[B24-jintelligence-13-00086] Fiske Donald W. (1949). Consistency of the factorial structures of personality ratings from different sources. The Journal of Abnormal and Social Psychology.

[B25-jintelligence-13-00086] Garnefski Nadia, Kraaij Vivian, Spinhoven Philip (2010). CERQ: Manual de Utilizare a Chestionarului de Coping Cognitiv- Emotional.

[B26-jintelligence-13-00086] George Darren, Mallery Paul (2010). SPSS for Windows Step by Step: A Simple Guide and Reference.

[B27-jintelligence-13-00086] Gertz Marlene, Schütz-Bosbach Simone, Diefenbach Sarah (2021). Smartphone and the Self: Experimental Investigation of Self-Incorporation of and Attachment to Smartphones. Multimodal Technologies and Interaction.

[B28-jintelligence-13-00086] Goldberg Lewis R. (1993). The structure of phenotypic personality traits. American Psychologist.

[B29-jintelligence-13-00086] Gonçalves Sónia P., Santos Joana Vieira dos (2022). Smartphone Use Side-by-Side with Burnout: Mediation of Work–Family Interaction and Loneliness. International Journal of Environmental Research and Public Health.

[B31-jintelligence-13-00086] Hair Joe F., Ringle Christian M., Sarstedt Marko (2011). PLS-SEM: Indeed a Silver Bullet. Journal of Marketing Theory and Practice.

[B32-jintelligence-13-00086] Hao Zejun, Jin Liangyi, Huang Jinzi, Akram Hafiza Rabia, Cui Qian (2023). Resilience and problematic smartphone use: A moderated mediation model. BMC Psychiatry.

[B33-jintelligence-13-00086] Hayes Andrew F. (2013). Introduction to Mediation, Moderation, and Conditional Process Analysis: A Regression-Based Approach.

[B34-jintelligence-13-00086] Horwood Sharon, Anglim Jeromy (2018). Personality and problematic smartphone use: A facet-level analysis using the Five Factor Model and HEXACO frameworks. Computers in Human Behavior.

[B35-jintelligence-13-00086] Hosťovecký Marian, Prokop Pavol (2018). The relationship between internet addiction and personality traits in Slovak secondary schools students. Journal of Applied Mathematics, Statistics and Informatics.

[B36-jintelligence-13-00086] Hussain Aftab, Ikram Ahmed, Aafreen Saima (2023). Relationship of smartphone addiction with cognitive and emotions regulation: An analysis among pre-school children. Pakistan Journal of Social Research.

[B37-jintelligence-13-00086] Hussain Zaheer, Griffiths Mark D., Sheffield David (2017). An investigation into problematic smartphone use: The role of narcissism, anxiety, and personality factors. Journal of Behavioral Addictions.

[B38-jintelligence-13-00086] John Nisha, Sahu Maya, Sharma Manoj Kumar, Murthy Pratima (2025). Personality Traits that Predispose or Protect in Smartphone Addiction and Their Implications for Intervention: A Narrative Review. Cyberpsychology, Behavior, and Social Networking.

[B39-jintelligence-13-00086] Kang Weixi (2022). Big Five personality traits predict illegal drug use in young people. Acta Psychologica.

[B40-jintelligence-13-00086] Kang Weixi (2023). Big Five personality predict epilepsy diagnosis in 7 years. Frontiers in Neurology.

[B41-jintelligence-13-00086] Kanwal Noreen, Isha Ahmad Shahrul Nizam (2022). The Moderating Effects of Social Media Activities on the Relationship Between Effort-Reward Imbalance and Health and Wellbeing: A Case Study of the Oil and Gas Industry in Malaysia. Frontiers in Public Health.

[B42-jintelligence-13-00086] Kheradmand Ali, Amirlatifi Elham Sadat, Rahbar Zahra (2023). Personality traits of university students with smartphone addiction. Frontiers in Psychiatry.

[B43-jintelligence-13-00086] Kim Byung-Jik, Kim Min-Jik, Lee Julak (2024). The impact of an unstable job on mental health: The critical role of self-efficacy in artificial intelligence use. Current Psychology.

[B44-jintelligence-13-00086] Kock Ned (2017). Common Method Bias: A Full Collinearity Assessment Method for PLS-SEM. Partial Least Squares Path Modeling.

[B45-jintelligence-13-00086] Koob George F., Le Moal Michel (1997). Drug Abuse: Hedonic Homeostatic Dysregulation. Science.

[B46-jintelligence-13-00086] Lai Daniel W. L., Li Jia (2022). Personality and health-related quality of life (HRQoL) of Hong Kong Chinese older people: Resilience as a mediator and financial status as a moderator. Aging and Mental Health.

[B47-jintelligence-13-00086] Lavi Gal, Rosenblatt Jonathan, Gilead Michael (2022). A prediction-focused approach to personality modeling. Scientific Reports.

[B48-jintelligence-13-00086] Lei Leonard Yik-Chuan, Ismail Muhd Al-Aarifin, Mohammad Jamilah Al-Muhammady, Yusoff Muhamad Saiful Bahri (2020). The relationship of smartphone addiction with psychological distress and neuroticism among university medical students. BMC Psychology.

[B49-jintelligence-13-00086] Linden Tanya, Nawaz Saqib, Mitchell Matthew (2021). Adults’ perspectives on smartphone usage and dependency in Australia. Computers in Human Behavior Reports.

[B50-jintelligence-13-00086] Liu Shiyu, Hu Wen, Yang Yingkai, Yang Fahui (2023). Body dissatisfaction and smartphone addiction: The mediation role of intrusive imagery and fear of negative evaluation. Frontiers in Psychology.

[B51-jintelligence-13-00086] Lyon Kieran A., Elliott R., Ware K., Juhasz G., Brown L. J. E. (2021). Associations between Facets and Aspects of Big Five Personality and Affective Disorders: A Systematic Review and Best Evidence Synthesis. Journal of Affective Disorders.

[B52-jintelligence-13-00086] Maddi Salvatore R. (1989). Personality Theories: A Comparative Analysis.

[B53-jintelligence-13-00086] McCrae Robert R., Costa Paul T. (1997). Personality trait structure as a human universal. American Psychologist.

[B54-jintelligence-13-00086] Minulescu Mihaela (2008). ABCD-M. Manual Tehnic şi Interpretativ.

[B55-jintelligence-13-00086] Moodie Craig A., Suri Gaurav, Goerlitz Dustin S., Mateen Maria A., Sheppes Gal, McRae Kateri, Lakhan-Pal Shreya, Thiruchselvam Ravi, Gross James J. (2020). The neural bases of cognitive emotion regulation: The roles of strategy and intensity. Cognitive, Affective, & Behavioral Neuroscience.

[B56-jintelligence-13-00086] Neal Zachary P., Brutzman Brian (2023). The role of personality in neighborhood satisfaction. PLoS ONE.

[B57-jintelligence-13-00086] Nikolic Aleksandra, Bukurov Bojana, Kocic Ilija, Vukovic Milica, Ladjevic Nikola, Vrhovac Miljana, Pavlović Zorana, Grujicic Jovan, Kisic Darija, Sipetic Sandra (2023). Smartphone addiction, sleep quality, depression, anxiety, and stress among medical students. Frontiers in Public Health.

[B58-jintelligence-13-00086] Niskier Sheila Rejane, Snaychuk Lindsey A., Kim Hyoun S., da Silva Thiago T., de Souza Vitalle Maria Sylvia, Tavares Hermano (2024). Adolescent Screen Use: Problematic Internet Use and the Impact of Gender. Psychiatry Investigation.

[B59-jintelligence-13-00086] Osorio Jacobo, Figueroa Marko, Wong Lenis (2024). Predicting Smartphone Addiction in Teenagers: An Integrative Model Incorporating Machine Learning and Big Five Personality Traits. Journal of Computer Science.

[B60-jintelligence-13-00086] Özbek Volkan, Alnıaçık Ümit, Koc Fatih, Akkılıç M. Emin, Kaş Eda (2014). The Impact of Personality on Technology Acceptance: A Study on Smart Phone Users. Procedia—Social and Behavioral Sciences.

[B61-jintelligence-13-00086] Podsakoff Philip M., MacKenzie Scott B., Lee Jeong-Yeon, Podsakoff Nathan P. (2003). Common method biases in behavioral research: A critical review of the literature and recommended remedies. Journal of Applied Psychology.

[B62-jintelligence-13-00086] Popescu Alexandrina-Mihaela, Balica Raluca.-Ștefania, Lazăr Emil, Bușu Valentin Oprea, Vașcu Janina-Elena (2022). Smartphone addiction risk, technology-related behaviors and attitudes, and psychological well-being during the COVID-19 pandemic. Frontiers in Psychology.

[B63-jintelligence-13-00086] Qiu Huake, Lu Hongliang, Wang Xianyang, Guo Zhihua, Xing Chen, Zhang Yan (2024). A moderated chain mediation model examining the relation between smartphone addiction and intolerance of uncertainty among master’s and PhD students. Heliyon.

[B64-jintelligence-13-00086] Ran Yaozong (2022). The Influence of Smartphone Addiction, Personality Traits, Achievement Motivation on Problem-solving Ability of University Students. Journal of Psychology and Behavior Studies.

[B65-jintelligence-13-00086] Sarhan Adnan Lutfi (2024). The relationship of smartphone addiction with depression, anxiety, and stress among medical students. SAGE Open Medicine.

[B66-jintelligence-13-00086] Seibert Daniel, Godulla Alexander, Wolf Cornelia (2021). Understanding How Personality Affects the Acceptance of Technology: A Literature Review. Media and Communication.

[B67-jintelligence-13-00086] Shahidin Siti Hajar, Midin Marhani, Sidi Hatta, Choy Chia Lip, Jaafar Nik Ruzyanei Nik, Sahimi Hajar Mohd Salleh, Roos Nur Aishah Che (2022). The Relationship between Emotion Regulation (ER) and Problematic Smartphone Use (PSU): A Systematic Review and Meta-Analyses. International Journal of Environmental Research and Public Health.

[B68-jintelligence-13-00086] Sindermann Cornelia, Riedl René, Montag Christian (2020). Investigating the Relationship between Personality and Technology Acceptance with a Focus on the Smartphone from a Gender Perspective: Results of an Exploratory Survey Study. Future Internet.

[B69-jintelligence-13-00086] Skoglund Tom Hilding, Brekke Thor-Håvard, Steder Frank Brundtland, Boe Ole (2020). Big Five Personality Profiles in the Norwegian Special Operations Forces. Frontiers in Psychology.

[B70-jintelligence-13-00086] Stada (2022). Share of People Who Experienced or Felt on the Verge of Burnout Europe in 2021, by Country [Graph]. Statista.

[B71-jintelligence-13-00086] Stanković Miloš, Nešić Milkica, Čičević Svetlana, Shi Zhuanghua (2021). Association of smartphone use with depression, anxiety, stress, sleep quality, and internet addiction. Empirical evidence from a smartphone application. Personality and Individual Differences.

[B72-jintelligence-13-00086] Statista (2021). Forecast of the Smartphone User Penetration Rate in Romania from 2015 to 2025 [Graph]. Statista.

[B73-jintelligence-13-00086] Statista (2024). Spending on Digital Transformation Technologies and Services Worldwide from 2017 to 2027 (in Trillion U.S. Dollars) [Graph]. Statista.

[B74-jintelligence-13-00086] Su Yunjing, Yan Zhonglian, Lin Wenqi, Liu Xuelin (2024). The Relationship Between Smartphone Addiction and the Interpersonal Competence of Chinese Private College Students: A Moderated Mediation Model. Psychology Research and Behavior Management.

[B75-jintelligence-13-00086] The Jamovi Project (2022). Jamovi. (Version 2.3) [Computer Software]. https://www.jamovi.org.

[B76-jintelligence-13-00086] Ting Chuong Hock, Chen Yoke Yong (2020). Smartphone addiction. Adolescent Addiction.

[B77-jintelligence-13-00086] Tripathi Anurag (2018). Impact of Internet Addiction on Mental Health: An Integrative Therapy Is Needed. Integrative Medicine International.

[B78-jintelligence-13-00086] Tu Wei, Nie Yangang, Liu Qingqi (2023). Does the Effect of Stress on Smartphone Addiction Vary Depending on the Gender and Type of Addiction?. Behavioral Sciences.

[B79-jintelligence-13-00086] Tudor Gabriel (2023). Proprietăți psihometrice ale versiunii în limba română a Smartphone Addiction Scale—Short Version (SAS-SV). Journal of Psychology/Revista de Psihologie.

[B80-jintelligence-13-00086] Tufan Cenk, Köksal Kemal, Griffiths Mark D. (2025). The Impact of Smartphone Addiction, Phubbing, and Fear of Missing Out on Social Cooperation and Life Satisfaction Among University Students. International Journal of Mental Health and Addiction.

[B81-jintelligence-13-00086] van Deursen Alexander J. A. M., Bolle Colin L., Hegner Sabrina M., Kommers Piet A. M. (2015). Modeling habitual and addictive smartphone behavior. Computers in Human Behavior.

[B82-jintelligence-13-00086] Wan Qunfang, Jiang Li, Zeng Yihua, Wu Xiaoling (2019). A big-five personality model-based study of empathy behaviors in clinical nurses. Nurse Education in Practice.

[B83-jintelligence-13-00086] Wang Ye, Ma Qianying (2024). The impact of social isolation on smartphone addiction among college students: The multiple mediating effects of loneliness and COVID-19 anxiety. Frontiers in Psychology.

[B84-jintelligence-13-00086] Wei Xinyi, Chu Xiaoyuan, Wang Hongxia, Geng Jingyu, Zeng Pan, Ren Lei, Liu Chang, Lei Li (2024). Does positive coping style alleviate anxiety symptoms after appearing problematic smartphone use for generation Z adolescents? The mediating role of state core self-evaluation. Current Psychology.

[B85-jintelligence-13-00086] Wiley (2024). Top Five Reasons Students Were Seeking More Mental Health Counseling or Therapy in North America as of 2023 [Graph]. Statista.

[B86-jintelligence-13-00086] Williams Matthew K., Waite Lennie, Van Wyngaarden Joshua J., Meyer Andrew R., Koppenhaver Shane L. (2023). Beyond yellow flags: The Big-Five personality traits and psychologically informed musculoskeletal rehabilitation. Musculoskeletal Care.

[B87-jintelligence-13-00086] Wrosch Carsten, Scheier Michael F. (2003). Personality and quality of life: The importance of optimism and goal adjustment. Quality of Life Research.

[B88-jintelligence-13-00086] Yin Xiangju, Yu Yongli, Qian Hongwei, Wang Zixu (2024). Family happiness and college students’ smartphone addiction control: The chain mediation effect of emotion regulation and self-control. Frontiers in Public Health.

[B89-jintelligence-13-00086] Yue Heng, Yue Xiwen, Zhang Xuemin, Liu Bo, Bao Hugejiletu (2022). Exploring the relationship between social exclusion and smartphone addiction: The mediating roles of loneliness and self-control. Frontiers in Psychology.

[B90-jintelligence-13-00086] Zhu Chunyue, Li Shuo, Zhang Lei (2025). The impact of smartphone addiction on mental health and its relationship with life satisfaction in the post-COVID-19 era. Frontiers in Psychiatry.

